# “The real pandemic’s been there forever”: qualitative perspectives of domestic and family violence workforce in Australia during COVID-19

**DOI:** 10.1186/s12913-022-07708-w

**Published:** 2022-03-15

**Authors:** Rachel Baffsky, Kristen Beek, Sarah Wayland, Janani Shanthosh, Amanda Henry, Patricia Cullen

**Affiliations:** 1 School of Population Health, UNSW Sydney, Samuels Building, F25, Samuel Terry Ave, Kensington NSW, Sydney, Australia; 2 Faculty of Medicine and Health, University of New England, Sydney Campus, Sydney, New South Wales Australia; 3 The George Institute for Global Health, UNSW Sydney, 1 King Street Newtown NSW, Sydney, Australia; 4 Australian Human Rights Institute, UNSW Sydney, Kensington NSW, Sydney, Australia; 5 School of Women and Children’s Health, UNSW Sydney, Kensington NSW, Sydney, Australia; 6 Ngarruwan Ngadju, First Peoples Health and Wellbeing Research Centre, University of Wollongong, Wollongong, NSW Australia

**Keywords:** Domestic violence, Family violence, Intimate partner violence, Abuse, Workforce, COVID-19

## Abstract

**Background:**

In 2020, Australia, like most countries, introduced restrictions related to the global pandemic of coronavirus disease 2019 (COVID-19). Frontline services in the domestic and family violence (DFV) sector had to adapt and innovate to continue supporting clients who were experiencing and/or at risk of DFV. There is a need to understand from the perspective of those on the frontline how DFV service responses in different contexts impacted their working conditions and subsequent wellbeing, and what they want to see continued in ‘the new normal’ to inform future effective practices. We address this by reporting on findings from in-depth interviews conducted with practitioners and managers from the DFV sector in Australia.

**Methods:**

Between July and September 2020 semi-structured interviews were conducted with 51 DFV practitioners and managers from a range of services and specialisations across legal, housing, health and social care services. The data was analysed using iterative thematic analysis.

**Results:**

The most common service adaptations reported were shifting to outreach models of care, introducing infection control procedures and adopting telehealth/digital service delivery. Adjacent to these changes, participants described how these adaptations created implementation challenges including increased workload, maintaining quality and safety, and rising costs. Impacts on practitioners were largely attributed to the shift towards remote working with a collision in their work and home life and increased risk of vicarious trauma. Despite these challenges, most expressed a sense of achievement in how their service was responding to COVID-19, with several adaptations that practitioners and managers wanted to see continued in ‘the new normal’, including flexible working and wellbeing initiatives.

**Conclusions:**

The pandemic has amplified existing challenges for those experiencing DFV as well as those working on the frontline of DFV. Our findings point to the diversity in workforce experiences and has elucidated valuable lessons to shape future service delivery. Given the continuing impacts of the pandemic on DFV, this study provides timely insight and impetus to strengthen the implementation of remote working and telehealth/digital support across the DFV sector and to inform better supports for DFV workforce wellbeing in Australia and other contexts.

**Trial registration:**

Not a clinical intervention.

## Introduction

When the coronavirus disease 2019 (COVID-19) was declared a pandemic on the 11^th^ of March, 2020, Australia, like most countries, responded with strict infection control measures [1]. These included physical distancing, remote working, self-isolation, quarantine and border closures [2]. Many people-facing businesses were forced to close in compliance with these restrictions, resulting in job and income losses for 28% of Australian families [3]. Schools closed, forcing children to learn remotely from home, and only children of essential workers, or children self-reported as at risk could attend. For the first time, most people and families were spending prolonged periods at home in this time of global crisis.

The COVID-19 pandemic was expected to increase the prevalence and severity of domestic and family violence (DFV) internationally, as seen after other crises such as earthquakes, bushfires and hurricanes [4, 5]. DFV encompasses a constellation of abusive behaviours that can include physical, sexual, emotional, financial and psychological abuse perpetrated by an intimate, or former intimate partner, or family member. DFV is a gendered form of violence with women experiencing DFV at far greater rates than men, and DFV is most often perpetrated by men [6]. DFV also intersects with other structural inequities such that those who experience intersecting axes of oppression, including racialised peoples, older people, children and young people and disabled people are more likely to have DFV perpetrated against them, the impacts are more complex and there are additional barriers to accessing appropriate support [7–10].

The pandemic is considered to be ‘the perfect storm’ for DFV because it amplifies multiple risk factors for DFV whilst also constraining the capacity of DFV services to appropriately respond [11]. The prevalence of violence was expected to increase as anxieties and fears generated by the pandemic, increased unemployment, food shortages, school closures, public health shutdowns and economic insecurity amplified significant risk factors such as poverty, mental and physical health and family conflict [12–14].

Surveys of DFV practitioners have reported that the demand for domestic and family violence services and complexity of cases increased during the early months of COVID-19 [15]. The United Nations interviewed practitioners from 69 countries and found evidence that the prevalence and severity of DFV increased globally as a result of COVID-19 restrictions [12]. Consistent with this, a survey of practitioners from Queensland, Australia reported their client numbers increased by almost 50% between March and May 2020 [16]. Ninety-one percent of these practitioners also reported their clients’ needs had become more complex as a result of COVID-19 [16]. DFV was considered more complex as COVID-19 restrictions created intersectional challenges with mental health, drug and alcohol use, housing and financial insecurities and barriers towards help-seeking and/or leaving abusive situations [16]. The increase in demand for services and complexity of cases was similar in a study of practitioners from Victoria, Australia [17, 18].

### DFV services responses

DFV services have had to develop innovative ways to respond to the changing nature of DFV during COVID-19 whilst still complying with public health orders. To overcome challenges with physical distancing restrictions, many DFV services have transitioned towards a digital mode of service delivery [12, 19–22]. Some services such as shelters cannot be delivered remotely and so continued to operate face-to-face [23]. Technology such as video calls, DFV hotlines, web chats, apps and text are being used to connect service providers to survivors of DFV remotely during lockdown conditions [19, 21, 22, 24, 25]. For example, Serbia has started a 24/7 SOS chat-based and e-mail support service [12].

Digital solutions can be delivered fee-free to some clients under various government and philanthropic funding models in Australia and internationally. They are low burden and scalable in terms of reaching victim-survivors who are not able to be supported face-to-face [21]. Practitioners from Australia have identified several benefits of the shift to online counselling including removing geographical, time and transport barriers to accessing services [16]. There was also evidence that men’s behaviour change programs in Queensland, Australia had transitioned online [16]. Male clients were able to continue to participate in their behaviour change programs remotely when face-to-face delivery was no longer possible, and online delivery removed the geographical and time constraints of face-to-face meetings [16]. Practitioners in the Netherlands also reported seeing fewer cancellations during digital service delivery compared to face-to-face and attributed this to the ease with which clients could access treatment [26].

Despite these benefits, telehealth/digital DFV service delivery presents several challenges both in a pandemic and generally. There are concerns that telehealth/digital service delivery can compromise women’s safety as it can leave a communication trail through which the perpetrator can see that the victim-survivor is seeking help [20, 21, 25]. Furthermore, there are risks for client privacy as it is harder for practitioners to ensure no one else sees their communication with the client and to store the clients’ correspondence in a deidentified way [25]. For example, some video chat software’s automatically store identifiable logs of calls between clients and practitioners. There are also concerns that some DFV services cannot be delivered online or to sufficient quality. For example, practitioners in the Netherlands reported how trauma therapy ceased as services shifted online because it was not considered safe to provide this therapy without the face-to-face support of a practitioner in the same room [26]. Practitioners in Queensland and Victoria, Australia, have expressed concerns that digital service delivery has negatively impacted their quality of service delivery as they find it harder to conduct risk assessments, make contact with clients, build rapport and follow up digitally [16, 17].

Lastly, there are concerns telehealth/digital DFV service delivery has reduced the availability and accessibility of support services for those who are most vulnerable due to inequities in digital participation. This ‘digital divide’ is due to barriers with internet access, affordability and digital capacity [27]. Concerningly, in Australia, groups with inhibited digital participation are also those most at risk of DFV, including low-income earners, people with low educational attainment, Aboriginal and Torres Strait Islander peoples, older adults and people living in remote or regional communities [27–29].

Shelters and other face-to-face essential services have responded to COVID-19 by introducing strict infection control measures such as extra cleaning and providing clients with PPE and personal sanitation packs [12] and cross-sectoral collaborations to meet the increasingly complex needs of clients during COVID-19 [16]. Shelters have had to limit the number of people to comply with physical distancing restrictions [12, 19]. However, many have provided pathways for survivors to access alternative temporary accommodation in hotels/motels to address this challenge [30].

### Impact of service adaptations on DFV practitioners

The increased demand for services coupled with changing service delivery has increased stress for practitioners in the DFV sector [16, 17]. A UN study found that staff are feeling stretched and overwhelmed as they work extended hours to meet the increasingly complex needs of their growing clientele [12]. The COVID-19 situation has made it challenging for frontline workers to take time off, putting them at risk of burnout [31], and some practitioners have already reported needing to take leave because of this [24]. There are also concerns that remote working will negatively impact mental health as they experience professional isolation and increased risk of vicarious trauma [23, 24]. Despite these widespread concerns, there has been limited investigation and insight into how practitioners perceive that service adaptations implemented in response to COVID-19 have been effective in supporting their clients and also their own wellbeing.

Thus, there is a need to understand from the perspective of those on the frontline how DFV service responses in different contexts impacted their working conditions and subsequent wellbeing, and what they want to see continued in ‘the new normal’ to inform future effective practices. We address this by reporting on findings from in-depth interviews conducted with practitioners and managers from the DFV sector in Australia. Our study was informed by three research questions:

1. How did DFV services adapt to the challenges of increased demand and social distancing restrictions in the early months of COVID-19?
2. What were the impacts of these adaptations on service delivery and workforce wellbeing?
3. What innovations do the workforce want to see continued?

## Methods

### Study design and ethics

Underpinned by pragmatism, we carried out an iterative thematic inquiry [32] to understand workforce perceptions of service adaptations and innovations in response to COVID-19 and the impacts of these changes on workforce wellbeing.

This study forms part of a wider project titled, ‘Responses to domestic and family violence during the COVID-19 pandemic’ funded under University of New South Wales Rapid Response Research initiatives. Ethics approval for this study was granted by the University of New South Wales Human Research Ethics Committee (HC200379). Reporting of our findings was informed by the consolidated criteria for reporting qualitative studies (COREQ).

### Research team and reflexivity

This study is situated within a broader program of research examining the sex and gender dimensions of COVID-19. The research team undertaking the project come to the study with learnt experience of DFV through an allied health, public health and medicine lens. Acknowledgment of assumptions as to how workforce might manage the stressful service delivery challenges of COVID-19, given the team itself were also experiencing similar challenges, were identified and agreement was reached as to how these assumptions could be set aside to allow the participant narratives to shape the data analysis using regular debriefing and reflexive analysis of the data collection process. This included using field notes throughout the data collection, which were used to shape our reflexive process.

### Recruitment

Purposive sampling was used to recruit practitioners and managers from a range of service types, and geographical locations, to enable a nuanced understanding of different service adaptations within the DFV sector across Australia. The research team identified and made contact with potential participants in the following two ways: 1) Organisations that previously indicated their support for the project were asked to send out a recruitment invitation email on the research team’s behalf and to post recruitment advertisements in suitable locations including e-newsletters and organisation’s social media pages; and 2) Workforce and peak body/government representatives were purposively identified and contacted through publicly available information and via research participants using snowball sampling. These potential participants were then contacted directly by the researchers via email.

Recruitment was ongoing from July 2^nd^ 2020- September 22^nd^ 2020. Participants were eligible for inclusion if they were employed by a service responding to DFV across health, community, legal or social services as a frontline worker; or as administrative, coordination or management staff; or if they were employed by a body or policy agency that has a role in DFV prevention and response.

### Context and setting

The DFV sector in Australia include services from legal, housing, police, welfare and health services such as primary health, mental health and hospitals. Not-for-profit DFV services are locally funded through government grants, corporate funding and/or philanthropy. Public DFV health services (such as hospital-based and community health services) are government funded. Private services (such as general practitioners and psychologists) are partially subsidised by Australia’s universal medical health insurance scheme (Medicare) with the gap paid by the client.

Interviews were conducted between 9^th^ July, 2020 – 30^th^ November, 2020. During this time, Victoria was the only Australian state in active lockdown. Other states had border closures, restricted domestic travel, physical distancing and many workplaces, including DFV services, encouraged remote working where possible. Interviews were conducted via telephone or a secure online video conference platform (Zoom or Microsoft Teams) depending on preference. The participant was either at home or at their workplace during data collection and the interviewer was working from home. No one other than the participant and interviewer was present on the call or video conference.

### Data collection

The interviews used a semi-structured interview guide developed by the research team. Participants were asked to reflect on the impacts of COVID-19 on their clients, themselves as workers and to identify what has been most effective in DFV response during the pandemic, including models of care, programs and service innovations. Interviews lasted between 20–90 min. All participants gave permission for their interviews to be voice recorded and transcribed. The decision to cease recruitment was informed by pragmatic considerations (resource constraints) and when we reached consensus that the data had yielded sufficiently useful and rich information, which was determined through our iterative analysis of the data [33].

### Characteristics of participants

Participants were drawn from across Australia, representing six states and the Northern Territory, with most working in New South Wales (57%). Participants represented a variety of services including DFV advocacy and crisis services (45%), health care (21%), court and legal support (18%) and counselling (16%), all responding to people experiencing DFV. Participants most commonly worked in services focussed on supporting women (49%). Participants also worked in services specialised in supporting Aboriginal and Torres Strait Islander peoples (12%), LGBTQI + communities (4%), older people (2%), men (4%), children and young people, (2%) and culturally and linguistically diverse communities (4%). Participants were almost all female (98%), and most commonly were managers or team leaders (45%) or frontline practitioners (37%). None of the participants who agreed to be interviewed dropped out or withdrew consent to participate.

### Data analysis

Interviews were analysed using iterative thematic analysis [32]. NVIVO software was used to manage and store the data (QSR International, 2020). Iterative analysis occurred in four phases. First, preliminary themes were identified based on the research team’s experience and knowledge of frontline service provision, as well as engagement with frontline service providers and rapid review of the literature describing the emerging challenges of service provision during the pandemic.

In the second phase, four authors (RB, PC, KB, SW) undertook line by line analysis of four transcripts and developed codes that mapped to the initial themes. Then RB and PC coded the first 10 transcripts together using a thematic framework, which was iteratively refined as needed. RB then coded the remaining 41 transcripts independently. The third phase involved checking, revising and adding to preliminary themes through data immersion. This involved RB, PC, KB and SW reviewing the coded data independently and coming together for three rounds of collaborative discussion to reach consensus on themes. Finally, we refined the list of key themes and clarified their meaning through group discussion and identified key data extracts. Potentially identifying information from illustrative quotes was removed and participants were assigned a non-identifying number for data extracts.

The findings were shared with participants through presentations in late 2020 and early 2021, and via a practitioner report that was emailed in February 2021. Participant feedback was consistent that the findings reported reflected their experience of delivering DFV support during the pandemic.

## Results

Thematic analysis revealed two distinct themes: 1) Awareness as to how COVID-19 created new implications for service delivery in Domestic and Family Violence and 2) Responsivity to, and addressing of, the impact on practitioners’ wellbeing when delivering services during a pandemic. Across these two themes, eight subthemes were identified (Table 1). What was revealed from the 51 participant transcripts was the need to understand how services adapted to the COVID-19 restrictions by co-constructing the commonalities that emerged across the participant reflections.

**Table 1:** Two themes and eight subthemes identified through iterative thematic analysis with exemplar quotes

Theme 1: Awareness as to how COVID-19 created new implications for service delivery in Domestic and Family Violence		Theme 2: Responsivity to, and addressing of, the impact on practitioners’ wellbeing when delivering services during a pandemic	
Increased workload: frontline workers on two pandemics	“I mean, we’re focusing on this pandemic, but the real pandemic’s been there forever, and it's not getting better” P16, manager, health care	The urgency was unrelenting and exhausting	“This work is relentless and overwhelming; and it’s true. Nothing has changed; it’s going to get worse.” P27, manager, health care
Maintaining high quality care	“That’s why we do what we do. It’s a human connection... and you’ve got to have that connection and you find a way” P30, counsellor, health care	Connection and disconnection	“We all agree that we miss the opportunity to debrief after a particularly heavy session.” P29, case worker, health care
Rising costs in the face of funding insecurity	“Once that funding is no longer available, we will go back to staff sitting on extremely high caseloads which means extremely high risk” P9, manager, DFV advocacy and crisis service	Blurring of personal and professional boundaries	“Talking about domestic violence to a client over a phone in your own home, it’s very different to having it in a workspace.” P38, manager, court support
Sense of achievement	“I don’t know what we could have done better” P9, manager, DFV advocacy and crisis service	Vicarious trauma and concern for what was to come	“It’s who we’re not seeing that worries me…” P6, manager, counselling

### Theme 1: awareness as to how COVID-19 created new implications for service delivery in domestic and family violence

Participants described a number of service adaptations, which largely centred on rapid implementation of telehealth/digital service delivery, extended outreach services and infection control (Table 2).

**Table 2:** Common DFV service adaptations to COVID-19 including outreach care, infection control, telehealth and digital support

Increased focus on outreach care	
As demand increased, services extended their hours. Some professionals went from working 9–5 to 24/7	“Most of our services have gone into a 24/7 contactable service which used to be more of like a nine to five service, just to ensure that- they [the clients] might be able to seek help.” P4, CEO, DFV advocacy and crisis service
Work spaces were adapted to minimise COVID-19 exposure for clients and staff. Home visits, outdoor meetings and welfare checks were used to connect clients to their community. Staggered office hours/days were also used to minimise contact between staff	“You can still go and knock on someone’s door and stand three metres back and check on their safety and wellbeing, especially in towns where we didn’t have phone numbers for them or they weren’t answering the phones.” P9, manager, DFV advocacy and crisis service
Some services partnered with charities to provide clients with access to practical resources such as food, housing and financial assistance to pay for utilities	“We brought in the [deidentified charity organisation]…to help with COVID because of the costs of living and stuff so we asked them to come to us and they set up a little office here once a week where people could just come and get their bills paid or emergency relief.” P28, manager, health care
Infection prevention	
As ‘essential services’, shelters were able to operate face-to-face during COVID-19 provided they adapted their policies to comply with public health protocols. Shelters introduced temperature checks and COVID-19 screening for clients and staff. Many were also limited in the number of clients they could accommodate	“All the policies have been changed around how we assess a client coming into the service, with their children. We've got a whole list of health questions that we need to ask… no one’s allowed to walk in the door unless they’ve been asked all the questions about, sick, all that, symptoms, hot spots. Temperature taken, the same with staff.. If they're not feeling well, they're sent home.” P13, manager, DFV advocacy and crisis service
Shelters increased the intensity and frequency of their cleaning and provided clients with hygiene packets including hand sanitizer, wipes and gloves. The wearing of personal professional equipment (PPE) was mandated among staff	“We increased the cleaning in our shelter. So we used to have fortnightly cleaning…but we now have weekly cleaning and we increased it from two hours to three hours. We give all the women a little pack with wipes and hand sanitizer, and we’ve taken masks to the shelter, and gloves. We had a dishwasher installed…to try and increase hygiene and infection control.” P5, executive director, DFV advocacy and crisis service
Telehealth/Digital services	
Most services other than shelters could no longer operate face-to-face. They adapted to deliver services remotely by telephone/digital platforms	“We’ve changed over to providing our services over the phone, and our playgroups and parenting support either through a Facebook group or the newsletter or by phone as well.” P12, manager, counselling “I do some group work via Zoom, so that works quite well. And generally my day to day work is telephone work.” P10, counsellor, DFV advocacy and crisis service
Legal services supported their clients to navigate video court appearances and online Applications for Apprehended Violence Orders (AVO)	“We've also been using things like Microsoft Teams or the court Zoom account for court appearances and so forth.” P44, Manager, court and legal support

The service innovations and adaptations created challenging and unprecedented changes to DFV service implementation during the early months of COVID-19. Four themes were identified that reflect participants’ experience of implementation challenges and their views on the way in which their service responded: 1) Increased workload: frontline workers on two pandemics, 2) Maintaining high quality care, 3) Rising costs in the face of funding insecurity and 4) Sense of achievement.

#### Increased workload: frontline workers on two pandemics

Participants emphasised the strain of responding to two pandemics; the ‘hidden’, ongoing DFV pandemic and the new COVID-19 pandemic. This was particularly stressful for participants who provided outreach services as they were at risk of being exposed to COVID-19 when checking in on clients at home. This risk was mitigated through practicing social distancing and handwashing, however still caused additional challenges for the health and wellbeing of frontline workers.

“We risk assessed around every individual case, I guess, and where individual contact was required, then we would do that, but we would just be mindful of social distancing practices and of hygiene.” P9, manager, DFV advocacy and crisis service.

Participants reported providing immediate and around the clock outreach services to address the multifaceted challenges with DFV unfolding in the early months of COVID-19. In remote communities, where resources were strained, DFV practitioners became first responders at DFV-related incidents. One participant reported an extreme case where her staff worked with police and other community members to intervene with a youth self-harm incident in the middle of the night.

“I have staff that work up until 3 am… so, right through to a response with [deidentified organisation] where community members had taken off up bush and were threatening suicide or attempting suicide up bush and staff would be driving around the bush looking for these young fellas… who were threatening or attempting suicide with police and other community members.” P9, manager, DFV advocacy and crisis service.

Service provision was also intensified by the switch from face-to-face to telehealth/digital delivery across most sectors. Counsellors reported that telehealth was associated with fewer cancellations, which was beneficial for the client, but increased the workload for the counsellor and limited their ability to take on new clients.

“Yeah, a lot of the outreach services, the counselling services, and even some of the shelters… switched to some forms of tele support as well...There were less cancellations of sessions. So that actually put a certain demand on the service, because the counsellors were all working at capacity and so they couldn’t take on new referrals.” P45, CEO, DFV advocacy and crisis service.

Participants reported how they were using the phone to contact clients more frequently than they would before the pandemic due to concerns for the elevated risks of DFV posed by the COVID-19 situation.

“We’re increasing our contact with clients, so that we’re trying to keep them in view and maintain engagement where we can. So, we’ve increased our contact to weekly or fortnightly, depending on the level of barriers.” P15, services coordinator, health care.

To support telehealth, participants reported providing more additional work outside of sessions, such as coordination emails and phone call follow ups which ultimately increased their workload.

“I do much more backup work as well to support them in terms of emailing and the occasional phone call just to see how they’re going and I do more of that. Whereas before it was more limited just to the sessions.” P29, case worker, health care.

In addition to transitioning their client support services online, most participants reported moving their internal meetings online. Many participants reported this increased their volume of meetings. They also reported they were not having breaks between meetings or the same opportunities to debrief as they would with face-to-face meetings.

“Well, one of things with Zoom, it makes it incredibly easy to have a lot of meetings in a day, like normally you… would drive somewhere and have a meeting and then you leave the meeting, get back in the car, drive back to the office, think about things, have a cup of tea; before we might have another meeting somewhere else. With Zoom, it’s incredibly easy to back-to-back them.” P45, CEO, DFV advocacy and crisis service.

Counsellors spoke about how they spent significant amounts of time addressing their clients’ concerns around COVID-19 in addition to providing regular therapy for their experience of DFV. In this sense, they experienced an increased workload as frontline workers on two pandemics.

“My staff started noticing that every time people either came in or by phone they would spend anything from 10 to 20 to 25 minutes just allaying their fears and anxieties about COVID so it added an extra basically half an hour of time for my counsellors in not doing therapeutic counselling but actually just listening.” P28, manager, health care.

#### Maintaining high quality care

Participants expressed concerns that the shift to telehealth/online support had a negative impact on the quality of service delivery. Many participants found it harder to build rapport and assess the severity of DFV or mental health risk via phone or online compared to face-to-face. They explained this was because they could not collect information about their clients from non-verbal cues such as body language. They expressed concerns they were missing vital information about their clients’ wellbeing which undermined their capacity to deliver appropriate care.

“Telephone counselling is very different to face-to-face counselling, because in face-to-face counselling you can see the person. You can look into their eyes. You read body language. You work with what they say and how they behave. So that is not happening with telephone counselling. So the process of counselling has intensified, because I am only having a voice to listen to. So sometimes I have to really put attention, a lot of effort into listening very carefully of what they – the tone of voice.” P29, case worker, health care.

“I, as a clinician, would rely heavily on body language… Now I’m having to purely rely on voice and their intonation and their pauses and background noises and all of that. So it is very difficult for assessments to be completed. We’re missing information.” P15, services coordinator, health care.

One service attempted to improve the quality of risk assessment via telehealth by developing a list of standardised questions to screen for mental health risks including suicidality over the phone. This screening tool was holistic and asked clients about their food and housing security, employment and financial circumstances which were likely affected by COVID-19.

“We put together a list of questions…for the facilitators to ask every man when they called, So on a scale of 1 to 10, how are your emotionally? And then we had how are you managing isolation…What’s happening in regards to your finance, your food.. What are the children doing to keep busy? Are you and your family at risk of homelessness? Are you frustrated with anything at present?” P20, manager, DFV advocacy and crisis service.

Participants also reported concerns that it was difficult to protect client’s privacy and confidentiality during telehealth/digital consultations when there is high-risk they are being monitored by a perpetrator during COVID-19. This was another way in which the shift to telehealth/digital support was perceived to undermine the quality of service delivery.

“Even if we were speaking with women directly it would often come out that it’s supervised, like we have overheard men in the backgrounds prompting them about what to say.” P11, counsellor, DFV advocacy and crisis service.

On the other hand, many participants explained how they would like to see the continuation of telehealth/digital options as they perceived these to be useful for some clients who have issues physically accessing services for reasons such as living remotely, living with a disability or health condition, or being in a high-risk situation. A community coordinator and a psychologist respectively reported:

“I think I will still maintain some phone and online appointments. I think that has worked for some people in terms of accessibility, in terms of not having to travel to my office and being able to access it… in a time that suits them. So, I think I am going to keep that as an option for… some of my longer-term clients who find that effective.” P48, social worker, counselling.

“I think the benefits that the clients have raised is it's opened up possibilities for people in regional and rural areas. It created a real sense of equality amongst folks.” P7, counsellor, counselling.

It was clear that many felt there is still a need for face-to-face interactions with clients as telehealth/online support did not work for all clients. For example, some clients from low socioeconomic backgrounds did not have the data to participate in services online.

“There isn’t any video, you know, Teams or Zoom or Skype. Clients don’t want it, interestingly. Some of them don’t have it. Our population we work with is predominantly the lower socioeconomic…they haven’t got much data.” P37, manager, DFV advocacy and crisis service.

Some older clients found it difficult to navigate digital services. Likewise, clients in crisis were often not in the right head space to navigate digital services.

“There’s the older clientele that have trouble navigating that kind of system, but even when someone’s in the head space of just going through a trauma and being in that flight or fight hypervigilance and they’re not really, I don't think, able to put plans like that”. P35, team leader, DFV advocacy and crisis service.

Whilst telehealth/digital support has accessibility benefits for some clients, participants felt these modes of service delivery can undermine the quality of service provision when delivered alone and are not accessible for people from low socioeconomic backgrounds, older adults, people with language barriers and/or issues with digital literacy or people experiencing coercive control or acute crisis.

#### Rising costs in the face of funding insecurity

Another commonly reported challenge associated with the service adaptations was the additional costs incurred for organisations. Participants working in shelters explained that introducing strict infection control procedures was expensive, and expressed frustrations that these costs were not being covered by additional government funding or subsidies.

“The increase in costs that we have absolutely… the amount of hand sanitiser…we’ve improved and increased our deep cleaning twice a week and that’s costing us a fortune and that’s not catered for in the budget that we get from [deidentified local government].” P28, manager, health care.

The switch to telehealth/digital support also bore a cost for DFV organisations. One participant explained how their service was providing lengthy telehealth consultations to their clients and that this was a challenge because of the cost of these calls.

“The cost of staff and those lengthy phone calls…some of the conversations were an hour, an hour and a half.” P20, manager, DFV advocacy and crisis service.

For many services, these rising costs were against the background of funding insecurity for their programs and staff, with many on short-term contacts and no certainty that these would be renewed. The issue of funding insecurity in the DFV sector existed pre-pandemic and is well documented, yet it was difficult for staff to reconcile being an essential worker in the pandemic and simultaneously not knowing if they would still be employed next month.

“We have continued processes of competitive tendering, so that causes massive disruption… So you’ve got all these great workers who are experienced who are trying to work out if they’ve got a job, are the conditions the same, do they have the same security, no they don’t, they’re losing entitlements” P17, executive, DFV advocacy and social services.

#### Sense of achievement

Despite acknowledging implementation challenges, participants consistently shared a sense of achievement in the way in which their organisation had adapted and innovated to continue to serve clients during COVID-19. There was a shared sense of pride in being solutions-focused in the early months of the pandemic and the innovations they had implemented.

“I don’t know what we could have done better… we’re pretty inventive in where we are and we look to fill gaps ourselves…people tend to put too many barriers up and not be solution-focused themselves. So we are pretty solution-focused.” P9, manager, counselling.

Almost all participants agreed that their organisation had succeeded in providing continued contact and support to their clients during the early months of COVID-19. Participants also reported that their organisation had successfully communicated information about rapidly changing policies and practices and implemented wellbeing initiatives which supported their capacity to deliver services in these challenging conditions. There were no notable differences in participants’ satisfaction with their organisational response by type of work, however, participants who were managers appeared to be more praising of their organisational responses compared to practitioners. Some practitioners occasionally raised suggestions for improvements, such as that their organisation could have resolved technological challenges more quickly or provided staff with better technology access and infrastructure.

“I think everybody just did the best that they knew how. We still provided services…I think we did quite a good job for still supporting women and letting them know that we’re still here for them… If a woman came into the centre in crisis, she didn’t get turned away, we still saw her, we just made sure it was at an appropriate distance and everything was cleaned.” P26, general practitioner, health care.

“I think the organisational response has been pretty good. We get very regular emails from the CEO with updates…They’ve (the organisation) set up some new wellbeing services and strategies which is all up… They sent around self-care packages and PPE packages. They brought out IT changes pretty quickly. I guess, IT’s the biggest difficulty that lots of people have probably faced, but I think the organisational response overall has been pretty good.” P47, social worker, DFV advocacy and crisis service.

### Theme 2: responsivity to, and addressing of, the impact on practitioners’ wellbeing when delivering services during a pandemic.

Participants’ perceptions of the challenges they faced adjusting to the increased service demand and changed service delivery were centred around four dominant themes: 1) The urgency was unrelenting and exhausting 2) Connection and disconnection, 3) Blurred personal and professional boundaries, and 4) Vicarious trauma and concern for what is to come.

#### The urgency was unrelenting and exhausting

Participants spoke about how they already did significant unpaid work as practitioners on the frontline of the DFV epidemic in Australia, but this workload was exacerbated by the added pressure of being an ‘essential worker’ during COVID-19 pandemic. Participants spoke about the added time it was taking to modify their usual policies and practices to comply with COVID-19 restrictions. They explained how this was particularly taxing for those who were also balancing learning from home as a consequence of school closures.

“I guess really it is that concern around workload…I think has been exacerbated during COVID. We always knew that workers in the sector did a lot of unpaid additional work, but again I think that’s a much harder thing to have a line of sight to when people are doing it from their home.” P17, executive, DFV advocacy and crisis service.

“It’s a very challenging time to be working as an essential service…Our staff have been working around the clock. We haven’t gone home. We’re here every day modifying what we need to do. How we need to work in adapting our ways that still prioritise safety, physical safety… And that’s a big ask for a workforce who are also trying to home school. Go home to their own families, worry about their own loved ones. Make sense of the world.” P50, director, DFV advocacy and crisis service.

Participants also reported that telehealth/online service delivery was more exhausting than face-to-face service delivery. It was not just the new medium of telehealth, but the length and volume of the phone calls that were draining for participants, creating fatigue for frontline service providers in the early months of COVID-19.

“It certainly was more taxing from when you’re on the phone and when you’re looking at a screen. Mind you, I did get used to the phone…That got easier. It’s just the screen time and everyone reported that, more tiring and everything takes longer.” P30, counsellor, health care.

“So it was really the length of the [phone] conversations. And we were exhausted. Every time we made a call, everyone’s going, “Not another phone call.”” P20, manager, DFV advocacy and crisis service.

There is a need to regulate meeting conditions, such as ensuring meetings are appropriately spaced out and within normal working hours to avoid over-extending practitioners. One participant explained how being in constant meetings during the early months of COVID-19 meant she was working extended days.

“I’m just constantly in different Zoom meetings dealing with different responses to COVID from the DFV sector… I usually start at seven in the morning, and so some days it would go through until five o’clock at night, not every day, but some days would be extra long days.” P45, CEO, DFV advocacy and crisis service.

There was also a very real fear of burnout in the face of new ways of working. Participants expressed concerns that they wouldn’t be able to sustain their extended workload with many attesting that they will need to exert boundaries to regulate the hours and intensity of their work.

“I can certainly see that I have been showing some signs of coming closer to burnout if I’m not careful and I do need to be more vigilant about that and stronger with my own boundaries.” P6, manager, counselling.

“We’re getting 40 to 50 calls a day…We are overwhelmed and that, of course, leads to the other elephant in the room, almost, is the pressure on the workforce and the very high risks of burnout and vicarious trauma that we’re all concerned with.” P27, manager, health care.

Practitioners in the DFV acknowledged they were at risk of burnout prior to COVID-19 due to being under resourced and overextended; COVID-19 has added a double layer to the existing DFV pandemic, which has overextended them even further.

#### Connection and disconnection

Participants reported finding it difficult to adjust to the social isolation associated with remote-working. Most participants reported missing the incidental interactions they had with their colleagues in the office. They reflected on how these incidental interactions made them feel socially connected and from a professional perspective, helped them resolve issues about complex or emotionally distressing cases. A social worker noted:

“You’re not really getting a lot of that incidental exposure to debriefing and exchange of information with your co-workers. I mean, obviously, you’d do that in a very formal setting at your regular meetings and you can make a phone call to a manager or another worker, but it’s really that incidental work, I think, that I found I really missed.” P49, social worker, counselling.

It is especially important for workers in the DFV sector to stay connected to their colleagues given the distressing nature of working with people who have experienced trauma. During COVID-19, practitioners reported that clients were experiencing increasingly severe trauma, and those working from home explained that they missed debriefing about these distressing cases with their colleagues, and without this support many felt they were experiencing vicarious trauma.

“We all agree that we miss the opportunity to debrief after a particularly heavy session. You know, or we just want to get an opinion where we might just say, have you got a minute? Or you might be having a coffee and it’s just, like, oh, my goodness, you won’t believe what just happened; and we have a confidential quick chat. And you can’t do that. So it means that you carry a lot of that. I would say there’s more vicarious trauma that’s going on because of the severity and the inability to do that. And I’ve experienced that and felt that.” P37, counsellor, health care.

Many participants reported how their organisation attempted to mitigate the isolating impacts of remote working on employees by encouraging regular, informal catch ups among staff via videoconference. Managers reported making a conscious effort to encourage staff to chat and share memes or funny images online as a way to boost morale.

“We started…using Microsoft Teams…between the team all working remotely. We also used that as a way of trying to feel connected to each other and still have a bit of a light-hearted moment between us to sort of lift everyone’s morale. Like, people might post a meme on there or something cute or something, just to sort of break the stress of the confronting work that we were all doing in a quite isolated way”. P12, manager, counselling.

Many participants felt the COVID-19 situation highlighted that some internal staff meetings could (and should) be delivered virtually as it is often unnecessary to meet face-to-face. Several participants also felt that connection with colleagues from other services actually increased through online meetings. There was also the advantage that online meetings enabled interagency collaboration. This was reported as critically important during this period of rapid change where it has been valuable to learn in real time what adaptations had and had not worked elsewhere. Participants wanted to see this continued beyond the pandemic for enhanced service collaboration and more online professional development. Again, there needs to be a balance between online and face-to-face meetings where possible to prevent practitioners from feeling professionally and socially isolated.

“Some meetings, like, regular meetings, we would have had in the past that we'd all have to meet at a building…I can’t see the benefit of travelling to the building anymore. If we can all do it online… I would hope that we can continue that as a service system and not have that half an hour drive” P34, team leader, DFV advocacy and crisis service.

“Other adaptions... Because we’re in [deidentified region] we have been able to engage more efficiently, I’d say, with counterparts that are across the state, so engaging more in the Zoom stuff has been really good from a regional point of view. More webinars, more communication and inclusion and access. That I would like to see continue.” P27, manager, health care.

#### Blurring of personal and professional boundaries

Participants spoke about the blurring of personal and professional boundaries they experienced whilst working from home, particularly if they had children who were learning from home, which was understandably challenging for maintaining client privacy and for their own wellbeing.

“When my five-year old’s home, it drives me mad, so I’m having to close the door and then I feel bad because I’m having to say to him, “I’m working.”… Other staff have reported the same thing is that having to say, not now, I’ve got to work.” P15, services coordinator, health care.

“I would go from talking with a woman about, you know, extreme violence and trauma and then step out the door and there would be my daughter there, right there, waiting for me and not having that boundary was really stressful and difficult for me, just having clients in a sense in my home, made it really difficult for me to have those barriers between work and home which I try really hard to keep in place as part of my self-care and taking care of myself in the work that I do. So, that was probably the biggest thing I struggled with.”, P48, social worker, counselling.

Indeed, there was the considerable collision of work and home life, and in many cases of working remotely there was a breaking down of the boundaries between work and home life. Practitioners from various service types who worked with their clients remotely from their home reported that it was more challenging to hear their client’s distress and manage their own distress in their home. There was no opportunity to leave their work behind and effectively their client’s stories lingered in what should have been their safe spaces, their homes.

“Because I’m doing it here it's like ‘it’ is in my house, the violence is in my house because I’m speaking and I’m hearing it, I’m trying to manage it in my home at my dining table so it just feels like it's here all the time.” P34, manager, court and legal support.

“I have like a studio apartment, so I was quite literally working and sleeping in the same place. And that really does take its toll. And I think boundaries is a really good way to explain that, that there is no boundary so when you’re in your space and you see something, and maybe you were looking at that particular thing – whether it’s a picture or whatever – whilst you were listening to someone’s very traumatic experience and that emotional content you’re then sort of triggered by that. And again you don’t have your colleagues to kind of debrief with. And that really makes it quite isolating as a worker.” P18, community connect worker, DFV advocacy and crisis service.

There were additional challenges during the learning from home period where staff were simultaneously managing their children’s home learning while working and supporting clients. One participant reported her staff having to resort to delivering services from the toilet because this was the only space where their children were not at risk of interrupting or overhearing sessions.

“(They) were trying to deliver their work by going into the bathroom and having to sit on the toilet to be able to know that they were in a space where the children weren’t coming and going and being potentially exposed to hearing the proceedings” P17, executive, DFV advocacy and crisis service.

There are also some benefits to flexible working that participants reported they would like to see integrated into regular practice. Many participants said they would like to see a mix of working from home and working in the office. A director of a shelter reported that she felt flexible working was inevitable going forward and that this would benefit working parents, carers and staff with additional needs.

“I don’t believe we’ll ever go back to having a service that says you can’t work from home…And I think that’s a good thing for flexibility, for parents and those that are carers, and those potentially with personal needs that require time out from being in a busy work place every day.” P50, executive, DFV advocacy and crisis service.

#### Vicarious trauma and concern for what is to come

It was reported that there are always risks of vicarious trauma for practitioners responding to DFV, but that these risks were amplified due to working remotely with less access to colleagues and less separation from work, and also because the measures that organisations typically put in place to protect workers were constrained in a work-from-home arrangement. For example, participants no longer had immediate access to supervision or the opportunity to incidentally debrief complex cases with their colleagues from home.

“Certainly we have a significant concern about increases to vicarious trauma that workers would be exposed to because of that change in service delivery, but also the measures that organisations put in place to protect workers from things like vicarious trauma are also strained in a work-from-home arrangement. So that ready access to immediate supervision and debrief.” P17, manager, health care.

Participants also explained how the challenges in separating their professional and personal lives put them at elevated risk of vicarious trauma during remote working, and that this was more of a risk for staff with less private space in their homes, for example, young practitioners who were more likely to live in share houses.

“That blurring of boundaries between your work environment and your home environment is another issue; I mean, it’s generally recognised that having a workspace, having a home space and having them separate is really important self-care measure, especially when workers are performing work that does expose them to things like vicarious trauma in this situation, so we are really concerned about that, and workers in this sector are… characteristically low paid workers. They don’t have a home office set up to be able to be able to perform their work, so they’re sitting in their kitchen or on the bed or in the bathroom and that then means long after your work shift finishes…that risk still continues to exist to the worker’s health and safety because of the trigger…inside their home environment.” P17, executive, DFV advocacy and crisis service.

Participants spoke of asking to come into the office a few days per week to mitigate the risks of vicarious trauma in a fully remote work model. One participant explained how her wellbeing improved once her service allowed her to switch from fully remote working to flexible working during COVID-19.

“Doing counselling out of your bedroom is not normal. It’s not okay. And I’m sleeping in the same place I’m hearing trauma, and that made it really, really complex…These young practitioners who are living in shared houses, working out of their bedrooms… so I had to push back around this is an occupational health and safety issue… So considering some people were able to come into the office and do a bit of photocopying and stuff, and I go, why can’t I work out of the office a few days a week? And then they let me. And I feel much better psychologically.” P10, manager, DFV advocacy and crisis service.

Many people spoke about their worries for the future, both for themselves, their colleagues but mostly for their clients. There was a sense that many of their clients had not been able to make contact and they worried for them and for what was to come: “It’s who we’re not seeing that worries me….” P6, manager, counselling.

A related fear was for particular groups of people. It is widely acknowledged that the impacts of the pandemic have not been experienced equally across society, which has shone a light on existing structural inequities. While women have been particularly impacted, the interviewees also identified the groups who have been disproportionately impacted, including people on temporary visas, older people, children and young people and people with disabilities.

“We’ve had a few women who are on temporary visas and we haven’t been able to get them into any sort of accommodation option at all. They’ve been declined temporary and affordable housing options because of their temporary visa status” P12, manager, counselling.

“At the height of the restrictions, there were 30% less child protection reports. Because, children weren’t going to school, and therefore weren’t under that surveillance” P16, manager, health care.

A common theme was that participants hoped the COVID-19 situation has raised awareness of the importance of initiatives to support the wellbeing of frontline workers in the DFV sector. A union representative explained how COVID-19 could be the catalyst to solve the longstanding problem of a lack of proper wellbeing support.

“I hope that there is a greater awareness of vicarious trauma now. I hope that there is a greater awareness around the need for supervision, and I mean professional supervision, not managerial supervision. That’s been a long ongoing problem in the sector that the union has had to battle with around the lack of appropriate supervision or not enough supervision” P17, executive, DFV advocacy and crisis service.

## Discussion

This study filled an important gap in understanding how COVID-19 service innovations and adaptations impacted the DFV workforce because of its comprehensiveness and national scale. The changes to working conditions as services introduced telehealth/digital services, infection control measures and extended outreach services created multiple challenges for maintaining quality care and workforce wellbeing. Despite a vastly increased workload, potential for vicarious trauma and rapidly changing conditions, the workforce shared a sense of achievement in continuing to support clients in what was essentially the frontline of two pandemics. Our findings point to several important learnings for DFV policy and practice both in Australia and elsewhere. The study, written during the second wave of COVID-19 in Australia during 2021, demonstrated the ways in which lessons from the first wave of COVID-19 may increase preparedness for future disaster responses both in Australia and internationally. The study was able to identify strategies that can be implemented to minimise burnout, vicarious trauma and support the wellbeing of the DFV workforce.

During the early months of COVID-19, DFV workforce experienced the compounding challenges of being frontline workers on two pandemics. Their workload increased to meet the heightened demand and they were providing more after-hours outreach services to clients in crisis. This is reinforced by findings from a study in the Netherlands [26] that reported that practitioners were overstretched and limited in their capacity to take on new clients during COVID-19. Given, this was a time where many people were experiencing DFV for the first time or an escalation of DFV [34], it was understandably stressful for services to be overstretched and unable to meet demand despite increased workload.

Practitioners also reported they were spending increased time supporting clients via telehealth/digital services, which they found more demanding compared to face to-face. Our study shows that the transition to telehealth/digital support created additional costs for DFV services in Australia. These additional costs have similarly been reported by services internationally as evidenced by a UN study across 69 countries [12]. Further, our study highlighted several challenges with the quality and safety of telehealth/digital services. Practitioners found it harder to build rapport, conduct risk assessments and protect client’s privacy via phone/online. These were the same concerns raised by frontline practitioners in Australia, United States and Europe, [16, 17, 21, 35], with concerns that it is not emotionally safe to conduct trauma-informed therapy online [21, 26]. Taken together with our findings, this demonstrates a need to identify mechanisms to enhance DFV telehealth/digital service delivery to ensure quality and safety for practitioners and clients.

The workforce also experienced ‘Zoom fatigue’ as a consequence of remote working and supporting clients via telehealth/digital services. ‘Zoom fatigue’ is a recently coined phenomenon, referring to the tiredness people report after spending too long on videoconferences. Recent research explains that videoconferencing is tiring because it creates excessive eye contact, shows people their own image which can be stressful, and creates additional cognitive load as people work harder to communicate without relying on gestures and non-verbal cues [36]. Our findings also show that participants experienced fatigue from back-to-back Zoom meetings, because they did not have opportunities to debrief as with face-to-face meetings. Not having opportunities to debrief is tiring for all workers, but is particularly problematic for DFV service providers who are at elevated risk of vicarious trauma. Organisations need to be mindful of ‘Zoom fatigue’ and consider a hybrid model for meetings where safe and possible to do so.

Despite the challenges faced, there was a unanimous sense of achievement among the DFV workforce for the way in which their services had innovated and adapted to continue to serve clients in the face of COVID-19. Whilst the workforce was proud of their innovation, it did take a toll on their wellbeing. Practitioners felt exhausted by their increased workload and the transition to COVID safe working. In particular, remote working was stressful for those who had their own children at home and were facilitating home learning. This reflected a blurring of the boundaries between personal and professional lives, and as anticipated, had a negative impact on wellbeing [23, 24]. This adverse impact of remote working is unsurprising given that the majority of the DFV workforce are women and the impacts of the pandemic have been disproportionately felt by women in terms of increases in carer responsibilities [12]. This collision of work and home life must be a key consideration going forward in the design and implementation of policies to support the DFV to work remotely.

Remote working was also challenging for staff in the sense that they were isolated from the usual mechanisms of workplace support. Consistent with prior research, our study showed that the workforce felt lonely and disconnected from their colleagues at times during remote working [17, 26]. There were concerns and shared experiences of increased vicarious trauma during COVID-19 among the workforce in our study. One possible explanation for this is that COVID-19 restrictions interfere with many factors that protect against vicarious trauma. Protective factors at the individual level include supportive colleagues, a team environment, work life balance and factors at the organisational level include debriefing meetings, having adequate physical space at work and a positive workplace culture [37, 38]. Our findings show that all of these protective factors were undermined during remote working, and that the impacts of this were not experienced equally across the workforce.

Younger and less experienced practitioners have been found to be most at risk of vicarious trauma in normal conditions [38]. In our study, younger practitioners were more likely to be living in shared housing without easy access to private space for remote working, placing them at further risk of vicarious trauma during remote working. Further research is needed to clarify which other groups of practitioners may be at elevated risk of vicarious trauma during crisis conditions so that appropriate supports and mitigation strategies can be put in place.

### Implications for policy, practice and research

Our study contributes to the growing body of evidence that the DFV workforce have been overloaded and exhausted in the early months of COVID-19. Understanding this problem marks the first empirical step towards developing evidence-based solutions to supporting workforce wellbeing. The changes to policy and practice that have been rapidly implemented in response to COVID-19 provide valuable learnings for improving DFV services and DFV workforce wellbeing both during the pandemic and beyond.

An important learning centres on the success that DFV organisations have had rapidly implementing a hybrid mode of service delivery with many services using telehealth/digital support to remain connected to their clients and colleagues. While there is strong support for continuing telehealth/digital support at least in a hybrid mode alongside face-to-face support, organisations need to be aware of who benefits and who does not from telehealth/digital support in terms of equitable access and unintended consequences. It is imperative that face-to-face support remains available for people who are marginalised by the digital divide, otherwise inequities accessing telehealth/digital services can increase risk and compound disadvantage. Moreover, our findings indicate that moving from face-to-face to telehealth/digital support can compromise quality and safety. Future research must include robust evaluation to inform best practice regarding this relatively new mode of service delivery. Importantly, future research should include victim-survivor perspectives to better understand who benefits from telehealth/digital services as well as to elucidate inherent inequities and implementation short-comings.

Alongside telehealth/digital services delivery, it is recommended that organisations continue to use new technologies to stay connected to their colleagues and peers from external networks. Video teleconference meetings can increase connectedness and be a great convenience, however in order to prevent “Zoom fatigue” it is important that meetings are appropriately spaced out with opportunities for breaks and debriefing.

In terms of workforce wellbeing, our study points to strong support from the DFV workforce for ongoing flexible working, which involves a blend of working remotely from home and working on site. While there are clear benefits, it is also essential to respond to the challenges for remote working that arise in the face of balancing work with caring responsibilities and periods of home learning. This means ensuring that staff have appropriately private space to conduct their client work, and have a safe home environment in which to work, with consideration given to ensuring safety for DFV workers who may also be at risk of DFV in their own home. It is essential that organisations incorporate remote working into their occupational health and safety policies to ensure that staff are appropriately supported and able to work effectively and safely.

The risk of vicarious trauma and burnout have been amplified for the DFV workforce during the pandemic. The majority of our participants were fatigued and held concerns for their current and future wellbeing. This emphasises the need to ensure timely access to clinical supervision, regular opportunities to debrief with colleagues when working remotely and to implement additional workforce measures such as paid leave and additional wellbeing support initiatives. Workforce wellbeing support initiatives are critical to safeguarding the workforce and mitigating the impacts of stress, vicarious trauma and burnout. A recent scoping review identified psychoeducation, mindfulness programs and art and recreational programs as interventions with evidence of effectiveness for reducing vicarious trauma, compassion fatigue and burnout [39]. These strategies could be trialled in the continuing COVID-19 context in which hybrid models of onsite and remote working are widespread. Further research, incorporating co-design with the workforce, is needed to develop and test strategies that mitigate these risks during the pandemic and beyond.

There is a well-documented need to provide additional funding during the pandemic for services to meet the increased demand for DFV support and to relieve the unsustainable workload of the current workforce [16]. Alongside this, cost-effectiveness needs to be considered when co-designing and trialling strategies to support workforce wellbeing, particularly given the scarcity of funding in the domestic and family violence sector both in Australia and internationally. Psychoeducation shows promise as a cost-effective strategy as it can be delivered to a large cohort of workers at the same time [40]. Mindfulness on the other hand, has been found to be more costly to deliver [41]. A recent study identified individual supervision as being more effective than group-based supervision at retaining staff in Australia’s DFV sector, however it is most costly [42]. Funders must consider how their funding constrains the type of supervision services can provide to workers, and make informed decisions about how to allocate resources to support worker wellbeing [42].

### Strengths and limitations

This study used a relatively large sample size compared to similar studies where samples varied from 16 to 40 [26, 43–46]. This enabled us to collect rich information about the diverse experiences of DFV workforce across Australia. Our purposive sampling strategy allowed us to recruit participants who were diverse in their geographical location, type of service provided and clientele served. We successfully recruited participants from six states/territories across Australia, which given that COVID-19 restrictions varied by state, allowed us to understand the breadth of workforce experiences. Further, our participants were drawn from diverse work contexts and worked with a range of client groups. Thereby, the findings from our study are relevant to a number of diverse contexts, including rural and remote communities.

We acknowledge that our final sample was overly representative of New South Wales, which is the most populous state in Australia. Recruitment involved use of professional networks, and the research team are based in New South Wales thus this likely influenced the recruitment of participants. It should also be acknowledged that participants who felt more strongly (either positively or negatively) about their experiences may have been more inclined to participate. Nevertheless, our study provides richly detailed insight into the experiences of the DFV workforce tasked with rapidly adapting to the conditions of the pandemic, and identifies valuable learnings for promoting safety and wellbeing of clients and staff as the pandemic continues to present challenges to responding to DFV globally.

## Conclusion

Through the perspectives of the workforce, our study demonstrates how COVID-19 has exacerbated existing challenges in the DFV sector. There have been profound implications for those working on the frontline of two pandemics, who face the challenges of under resourcing, fatigue and risks of vicarious trauma. This reflects the reality for frontline DFV workforce globally who have been working on the frontlines of two pandemics for almost two years and are at high risk of burnout. To counteract these risks, there is a critical need for robust evaluation to determine what service innovations have worked well for both clients and the workforce since the start of the pandemic, and what are the unintended consequences and implementation shortcomings. Lastly, we must think beyond COVID-19 and consider how new technologies can be effectively harnessed to facilitate connectedness between services, their clients and the workforce.

## Data Availability

The datasets used/and or analysed during the current study are not publicly available in accordance with requirements approved by the University of New South Wales Human Research Ethics Committee. For general queries about data collection procedures and materials, please contact the corresponding author, Dr Patricia Cullen: patricia.cullen@unsw.edu.au.

## Abbreviations

COVID-19: Global pandemic of coronavirus disease 2019
DFV: Domestic and family violence
AVO: Apprehended Violence Orders
PPE: Personal protective equipment
